# Establishment and application of a root wounding–immersion method for efficient virus-induced gene silencing in plants

**DOI:** 10.3389/fpls.2024.1336726

**Published:** 2024-04-19

**Authors:** Xinyun Li, Na Tao, Bin Xu, Junqiang Xu, Zhengan Yang, Caiqian Jiang, Ying Zhou, Minghua Deng, Junheng Lv, Kai Zhao

**Affiliations:** ^1^ Key Laboratory of Vegetable Biology of Yunnan Province, College of Landscape and Horticulture, Yunnan Agricultural University, Kunming, Yunnan, China; ^2^ College of Agronomy and Biotechnology, Yunnan Agricultural University, Kunming, China

**Keywords:** root wounding–immersion method, virus-induced gene silencing, agroinoculation, tobacco rattle virus, gene function

## Abstract

In the post-genomic era, virus-induced gene silencing (VIGS) has played an important role in research on reverse genetics in plants. Commonly used *Agrobacterium*-mediated VIGS inoculation methods include stem scratching, leaf infiltration, use of agrodrench, and air-brush spraying. In this study, we developed a root wounding–immersion method in which 1/3 of the plant root (length) was cut and immersed in a tobacco rattle virus (TRV)1:TRV2 mixed solution for 30 min. We optimized the procedure in *Nicotiana benthamiana* and successfully silenced *N. benthamiana*, tomato (*Solanum lycopersicum*), pepper (*Capsicum annuum* L.), eggplant (*Solanum melongena*), and *Arabidopsis thaliana* phytoene desaturase (*PDS*), and we observed the movement of green fluorescent protein (GFP) from the roots to the stem and leaves. The silencing rate of *PDS* in *N. benthamiana* and tomato was 95–100%. In addition, we successfully silenced two disease-resistance genes, *SITL5* and *SITL6*, to decrease disease resistance in tomatoes (CLN2037E). The root wounding–immersion method can be used to inoculate large batches of plants in a short time and with high efficiency, and fresh bacterial infusions can be reused several times. The most important aspect of the root wounding–immersion method is its application to plant species susceptible to root inoculation, as well as its ability to inoculate seedlings from early growth stages. This method offers a means to conduct large-scale functional genome screening in plants.

## 1 Introduction

Virus-induced gene silencing (VIGS) is a reverse genetics tool for effectively silencing target gene expression by using plant antiviral defense mechanisms that inhibit invading viruses (Dommes et al., 2019). Currently, most functional gene research methods, including chemical mutagenesis, T-DNA insertion, and CRISPR/Cas, are dependent on stably transformed lines and generally require labor-intensive processes such as phenotyping and molecular screening, and several of the methods are inefficient. In some cases, the loss of function of some essential genes causes plant death during the early stages of plant growth, which may limit the application of these methods (Baulcombe, 2004; Benedito et al., 2004). In comparison, VIGS is a low-cost and rapid tool, and a large number of plant viruses have been successfully developed as VIGS vectors, including tobacco rattle virus (TRV), tobacco mosaic virus (TMV), potato virus X (PVX), tomato golden mosaic virus (TGMV), and cabbage leaf curl virus (CbLCV) (Burch-Smith et al., 2004).

Modified VIGS systems have been used in Brassicaceae (Fridborg et al., 2013; Wang et al., 2013; Yu et al., 2019), Solanaceae (Fu et al., 2005), Gramineae (Bennypaul et al., 2012; Kang et al., 2013; Martin et al., 2013), Cucurbitaceae (Bu et al., 2019; Liao et al., 2019), Asteraceae (Deng et al., 2012), Leguminosae (Ganiger et al., 2013; Atwood et al., 2014), Orchidaceae (Hsieh et al., 2013a, Hsieh et al., 2013b), and Malvaceae (Gao et al., 2011; Pang et al., 2013). The silencing efficiency of VIGS is related to the inoculation method, bacterial suspension concentration, vector selection, and environmental conditions. Virus vector-induced silencing efficiency is associated with host selection. Currently, *Agrobacterium*-mediated inoculation is mainly used for VIGS, and the primary methods for this type of inoculation are injection (Huang et al., 2014), agrodrench (Ryu et al., 2004), high-pressure spray (Liu et al., 2002a), and vacuum infiltration (Yan et al., 2012). TRV is widely used in VIGS vector construction owing to its high silencing efficiency, long silencing duration, and mild infection symptoms (Dinesh-Kumar et al., 2003). Studies have confirmed that low temperature and low humidity can increase VIGS silencing efficiency (Fu et al., 2006; Singh et al., 2018; Yang et al., 2018), and TRV-VIGS inoculated through agrodrench application or leaf infiltration can persist for 2 years or more (Senthil-Kumar and Mysore, 2011a). In agricultural inoculation, the concentration of the infiltration solution also considerably affects gene silencing in VIGS experiments; for example, an OD600 > 1 causes *Nicotiana benthamiana* leaf necrosis. In comparison, the OD600 = 1.5 of *Agrobacterium* results in good infection effects in tomatoes (Senthil-Kumar et al., 2007).

Several Agrobacterium-mediated inoculation methods have been developed to introduce VIGS and other related applications, such as virus-mediated genome editing (Ali et al., 2015), into host plants. Among these methods, root inoculation is a promising approach that can be applied to research on root systems and on plant hosts that are resistant to above-ground infection, as well as in other similar cases. TRV vectors can infect plant roots and efficiently express green fluorescent protein (GFP) (MacFarlane and Popovich, 2000). In addition, one study found that a modified TRV vector showed stronger infectivity and invasiveness in meristems in the rhizosphere than elsewhere (Valentine et al., 2004). The present study employed a root wounding-immersion method to trigger VIGS with TRV vectors. This method is suitable for *N. benthamiana*, tomato (*Solanum lycopersicum*), eggplant (*Solanum melongena*), pepper (*Capsicum annuum* L.), and *Arabidopsis thaliana*. This study provides a new VIGS inoculation approach for functional research on plant genes.

## 2 Materials and methods

### 2.1 Plant materials and growth conditions

Tomato (Micro Tom and CLN2037E), *Arabidopsis thaliana*, *Nicotiana benthamiana*, eggplant (S-34), and pepper (*Capsicum annuum*) were all stored by our group. The seeds were placed in cell culture dishes with moist tissue paper and covered, after which they were incubated at 25°C under high humidity for 3 days to stimulate germination. After breaking through the testa, the plants were cultured indoors with 16 h (28°C) of light and 8 h of darkness (20°C).

### 2.2 Plasmid construction

**Figure 1:**
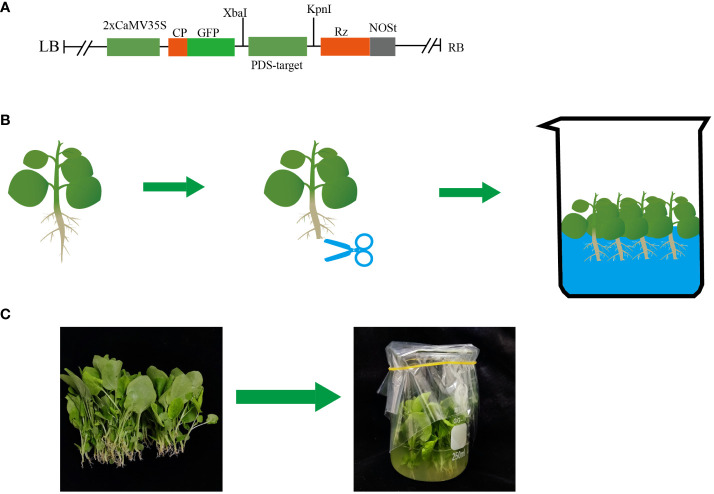
Schematic diagram of the root wounding–immersion method. **(A)** Construction of the pTRV2-*GFP* backbone; **(B, C)** Schematic of the root wounding–immersion process.

pTRV2-*GFP* was used as a backbone (**Figure 1A**) and *GFP* and phytoene desaturase (*PDS*) were used as reporter genes. Ryu et al. (2004) found that TRV2-NbPDS could silence endogenous PDS homologs in tomato, tobacco, and Petunia, showing that the *PDS* gene sequences of these species were conserved. However, TRV2-NbPDS did not inhibit the PDS homologs in pepper, potato, or eggplant (Ryu et al., 2004). To ensure accurate targeting in different hosts, we designed specific primers for different species to clone 300 bp of *PDS*, *SITLP5*, and *SITLP6* silencing fragments, which were ligated to the binary vector TRV2-*GFP* to generate TRV2-*GFP*-*NbPDS*, TRV2-*GFP*-*SlPDS*, TRV2-*GFP*-*CaPDS*, TRV2-*GFP*-*SmPDS*, TRV2-*GFP*-A*tPDS*, TRV2-*GFP*-*SITLP5*, and TRV2-*GFP*-*SITLP6*. The designed primers are shown in **Supplementary Table S1**. Electroporation was used to transform *Agrobacterium* GV1301 using the binary vectors pTRV2-*GFP*-*PDS* and pTRV1.

### 2.3 Development of root wounding–immersion method and *GFP* imaging

*Agrobacterium* GV1301 containing pTRV2-*GFP*-*PDS* and pTRV1 were cultured on LB plates containing kanamycin (50 μg/mL) and rifampin (25 μg/mL) at 28°C for 2 days. Positive colonies were selected and cultured overnight in LB broth containing the corresponding antibiotics and shaken at 200 rpm. A NanoDrop 1000 spectrophotometer (Thermo Fisher Scientific, New York, NY, USA) was used to determine that the OD600 of the inoculum was >1.

One day before VIGS, 50 μL of the above culture was added to 20 mL of LB medium containing 50 μg/mL kanamycin, 25 μg/mL rifampin, 20 μM acetosyringone, and 10 mM MES and cultured overnight at 28°C and 200 rpm. The following day, an infiltration solution (10 mM MgCl_2_, 10 mM MES at pH 5.6, 150 μM acetosyringone) was used to resuspend the culture until OD = 0.8. TRV1 and TRV2-carrying *Agrobacterium* suspensions were cultured in the dark at 28°C for 3 h (Zhang and Thomma, 2014; Meziadi et al., 2016). For the root wounding–immersion procedure, seedlings were first cultured in a tray. To increase the resistance of the plants, seedlings with 3–4 real leaves that were 3 weeks old were removed from the soil (including the roots). After pure water was used to remove soil and other impurities from the roots, a disinfected leaf knife was used to remove 1/3 of the root lengthwise. We designed two different immersion protocols: 1) “ concurrent inoculation,” where TRV1 and TRV2 were mixed and the plant was immersed for 30 min; and 2) “successive inoculation,” where the plant was immersed in TRV1 infiltration solution for 15 min and then transferred to a TRV2 infiltration solution for 15 min of immersion. To determine the effect of temperature on infection efficiency, the cut seedlings were placed in infiltration solutions with different temperature gradients in the two immersion protocols so that the roots were completely immersed. Shaking was carried out every 5 min. After 30 min, the seedlings were transferred to a 50-pore plastic tray containing sterilized soil (peat and vermiculite). After 48 h of dark treatment, the plants were cultured indoors with a 16-h (temperature was the same as immersion) light and 8-h dark (18°C) cycle with 35% humidity. Furthermore, we designed experiments with different concentrations and multiple uses of the inoculum. A fresh inoculum of 10 mL was prepared to inoculate 20 tomato plants simultaneously, using the same infection methods as described. A portable light source (LUYOR-3410RB, Luyor, USA) was used for *GFP* imaging.

### 2.4 RNA isolation and RT-qPCR analysis

To measure the silencing efficiency in TRV-infected plant leaves, a Huayueyang rapid universal plant RNA extraction kit (Huayueyang Biotechnology Co., Ltd., Beijing, China) was used to extract the total RNA from the leaves 30 days after infection. The quality and quantity of the extracted RNA were confirmed by 1.5% (w/v) agarose gel electrophoresis and a NanoDrop 1000 spectrophotometer (Thermo Fisher Scientific, New York, NY, USA). Then, 2 µg of total RNA was used to synthesize cDNA based on the manufacturer’s instructions for the TransScript one-step gDNA removal and cDNA synthesis SuperMix (TransGen, Beijing, China) kits. Primer Premier 6 (Premier, San Francisco, CA, USA) software was used to design specific primers for the segments outside the silencing regions. The species-specific internal reference genes were used as control and the primers were synthesized by Tsingke Biotechnology (Qingke Biotechnology Co., Ltd., Beijing, China). SYBR Green PCR Master Mix (TransGen) was used for RT-qPCR on a CFX96 (Bio-Rad, Hercules, CA, USA). Relative mRNA transcript abundance was calculated for TRV-GFP- and TRV-GFP-PDS-infected plants using the 2^−ΔΔCt^ method (Zhao et al., 2013) with uninfected plants as controls. All of the primers used are shown in **Supplementary Table S1**.

### 2.5 Pathogen inoculation and disease resistance identification

The late blight pathogen (*Phytophthora infestans*) was obtained from the Institute of Vegetables and Flowers at the Chinese Academy of Agricultural Sciences (Kunming, Yunnan Province, China). Then, 5 × 10^4^ sporangia/mL was used to spray six or seven leaves of VIGS-successful and control tomato seedlings. After inoculation, the seedlings were placed in a plant growth room, and 100% relative humidity (RH) and 20 ± 1°C were maintained. The seedlings were grown in the dark for 24 h. Then, the seedlings were grown under a 14 h/10 h light/dark cycle with a relative humidity of 60–80% (Jiang et al., 2018). Seven days after inoculation, the method described by Wang (2003) was used to calculate the disease severity rating (DSR), which was used as a reference for the resistance level.

### 2.6 Chlorophyll content measurement

The chlorophyll measurement was immediately performed after the extract was prepared based on the method of Hajirezaei et al. (2002). Then, 50 days after infection, the wild type was used as the control, and 50 mg of plant leaves were harvested and frozen in liquid nitrogen. Thereafter, 0.5 mL of 80% (v/v) acetone/water was used for extraction before 0.5 mL of 100% acetone was used for extraction to ensure that chlorophyll a and b were completely extracted. The samples were incubated at 80°C for 60 min, and spectrophotometry was used to measure the total chlorophyll, chlorophyll a, and chlorophyll b at 652, 647, and 664 nm, respectively.

### 2.7 Phytoene analysis

The samples were rinsed with ice-cold PBS (0.05 mol/L Tris-HCI, pH 7.4 phosphate buffered saline), and filter paper was used to remove the PBS. Then, 0.1 g of each sample was placed in 5 mL of homogenization tubes. Nine volumes of homogenization medium were added to the homogenization tube based on weight (g):volume (mL) = 1:9. The pestle was moved up and down 10 times in an ice bath to prepare 10% homogenate. Afterward, phytoene was measured using the plant phytoene ELISA Kit (Xingtai Sinobest Biotech Co., Ltd.) according to the manufacturer’s instructions.

### 2.8 Statistical method

Each experiment contained at least three biological replicates. IBM® SPSS® Statistics 24 statistical software (IBM, Chicago, IL, USA) was used for data processing. One-way ANOVA was used for data analysis, and Duncan’s test was used for post-hoc analysis. A p ≤ 0.05 was considered significant, and the results are presented as the means ± SD.

## 3 Results and discussion

### 3.1 Optimization of the root wounding–immersion procedure

**Figure 2:**
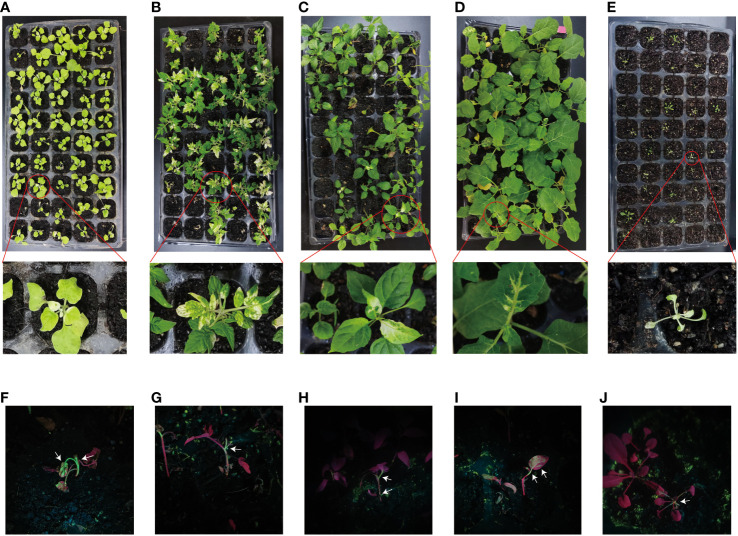
The VIGS system established through the root-soaking method resulted in plant bleaching. **(A–E)** Bleaching symptoms photographed in *Nicotiana benthamiana*, tomato, pepper, eggplant, and *Arabidopsis thaliana*, respectively, on day 30; **(F–J)** Green fluorescence of *N. benthamiana*, tomato, pepper, eggplant, and *A. thaliana* on days 7, 7, 10, 12, and 4, respectively.

Current inoculation methods mainly include *Agrobacterium*-mediated injection infiltration and vacuum infiltration. However, injecting *Agrobacterium* cultures into the seedling leaves of plants with tough tissues, such as soya beans and maize, can be challenging. Additionally, the leaves of these plants need to be fully unfolded to ensure successful injection (Ratcliff et al., 2001). For roots that are susceptible to inoculation and early-growth seedlings, we developed an inoculation method known as root wounding–immersion (**Figure 1B**). To test the feasibility of the root wounding–immersion method, we chose PDS as a reporter gene because the leaves of plants in which PDS is silenced tend to show symptoms of photobleaching (Kumagai et al., 1995; Liu et al., 2002a). To visualize TRV viral transport from the roots to the above-ground parts of plants, we cloned tobacco *NbPDS* fragments and inserted them into the vector pTRV2-*GFP*. Uninjured tobacco was infected according to the method described in Section 2.3 and the silencing rate was found to be less than 1%, so we used uninoculated plants as controls. The frequency of VIGS was defined as the number of plants that show silencing phenotype (photobleaching) after inoculation with TRV2-*GFP-NbPDS*. In *N. benthamiana*, the ratio of positive silenced plants was 95.8%, and *GFP* insertion did not modify the gene silencing capacity of the TRV vector (Tian et al., 2014). Under illumination with the portable excitation light source (LUYOR-3410RB), pTRV2-*GFP*-*NbPDS* was transferred from the roots to the stem and leaves (**Figure 2F**). We designed two different infection methods for different temperatures (**Supplementary Table S1**): “concurrent inoculation” and “successive inoculation.” Photobleaching was observed in *N. benthamiana* plants as early as day 6 (**Figure 2A**), which was faster than the 7–10 days reported in the study of Ryu et al. (2004).

The data show that the survival rate and silencing rate of concurrent inoculation were slightly higher than those for successive inoculation. For example, at 22°C, the survival and silencing rates of concurrent inoculation were both 100%, whereas the survival and silencing rates of successive inoculation were 96% and 92%, respectively. We believe the separate immersion in the TRV1 and TRV2 bacterial suspensions decreased opportunities for both to contact each other, which may decrease endogenous RNA-dependent RNA polymerase (RDRP) activation and the replication and production of virus dsRNA (Donaire et al., 2008). In addition, decreased TRV1 and TRV2 bacterial suspension immersion duration also decreased opportunities for entry into the plant through the wounded root.

Temperature is a key factor affecting the development of the gene-silencing phenotype of VIGS plants (Szittya et al., 2003; Fu et al., 2006; Tuttle et al., 2008), as most viruses lose their potency, decreasing virus concentration. However, low temperatures (16–21°C for tomatoes) can promote virus silencing (Szittya et al., 2003; Singh et al., 2018). Our data show that an appropriate temperature is beneficial for maximizing VIGS silencing effects. In concurrent inoculation, photobleaching occurred after 6 days at 22°C and after 12 days at 26°C, and VIGS effects were delayed. The survival and silencing rates are also affected by temperature. The survival and silencing rates of concurrent inoculation at 18°C were 72% and 79%, respectively, and the survival and silencing rates at 26°C were 60% and 56%, respectively. Because the effect of concurrent inoculation was better than that of successive inoculation, concurrent inoculation was adopted in the following studies.

A suitable concentration of permeable solution is beneficial to gene silencing efficiency in VIGS experiments. The most suitable infiltration solution concentration for *N. benthamiana* is OD600 = 1.0 because an inoculum with OD600 > 1.0 may cause leaf necrosis in *N. benthamiana* (Caplan and Dinesh-Kumar, 2006), and Dinesh-Kumar et al. (2003) found that OD600 = 1.5 shows better results in tomatoes. Burch-Smith et al. (2006a) found that the efficiency is almost 100% when the *A. thaliana* Col-0 ecological TRV-VIGS experiment concentration is OD = 1.5. It is necessary to determine the most favorable TRV-VIGS inoculation concentration, and we used different concentrations for concurrent inoculation at 22°C for *N. benthamiana* inoculation (**Table 1**). We found that the survival rate and *PDS* silencing effectiveness are dependent on the concentration of the *Agrobacterium* suspension. When OD = 0.4–1.2, the rate of *PDS* silencing was more than 95%. However, the *PDS* silencing rate decreased when the concentration was below 0.4, and necrosis occurred when the concentration was above 1.2. Therefore, we resuspended the bacteria to OD600 = 0.8 in the following studies to ensure a higher survival rate and silencing efficiency while considering the efficient use of the *Agrobacterium* resuspension.

**Table 1:** Optimization of concentrations for the root wounding–immersion method.

Concentration (OD600)	Time of onset of photobleaching (days)	Survival rate (%)	Silencing rate (%)
0.1	6	97	85
0.2	6	95	91
0.4	7	95	98
0.6	6	97	98
0.8	6	100	100
1.0	7	97	98
1.2	6	91	96
1.4	6	86	89

Even though root inoculation was performed in the root wounding–immersion, the movement of virus to aboveground organs was not delayed in *N. benthamiana*. In contrast, we found that the root wounding–immersion method does not require leaf infiltration for every *N. benthamiana* plant, and batch processing can be carried out. This method is highly efficient. In addition, we used our previous bacterial suspension (guaranteed fresh) many times. Results showed that the silencing rate was more than 90% (**Supplementary Table S2**) even after the TRV1:TRV2 mixed bacterial suspension was used five consecutive times. This avoids the preparation of a large volume of infiltration solution for the treatment of a large quantity of materials and saves time and costs. Our method confirms the hypothesis of Ryu et al. (2004) that *Agrobacterium*-mediated transformation occurs when there is simulated wounding to roots in growing plants.

### 3.2 Application of the root wounding–immersion method in *A. thaliana*, tomatoes, eggplants, and pepper

Infection was carried out using concurrent inoculation at a concentration of OD = 0.8 and 22°C (**Supplementary Table S3**). We found that the root wounding–immersion method is similarly effective in these five plant species, but the infection efficiency differed between different plant species (**Figures 2B–E**). TRV typically causes mild symptoms within 10 days of inoculation in *N. benthamiana* and tomatoes (Liu et al., 2002a). The virus spreads from the inoculation site to the developing regions of the plant and triggers PTGS. At 2–3 weeks, the virus spreads to the completely open upper leaves (Burch-Smith et al., 2004). Using the root wounding–immersion method, we found that photobleaching occurred 1 week after inoculation. The silencing rate of the tomatoes was 95% (**Supplementary Table S3**). TRV-mediated VIGS silencing has similarly been used in peppers. Generally, the pale yellow (*rbcS*) and photobleached leaf (*PDS*)-silenced phenotype will become significant 2 weeks after TRV vector transformation (Chung et al., 2004). We employed the root wounding–immersion method, and these phenotypes occurred 24 days after inoculation in peppers. The silencing rate of the tomatoes was 58% (**Supplementary Table S3**). Liu et al. (2012) used high-pressure spraying and injection on eggplant plants, and uniform bleaching occurred in the newly formed leaves after 30 days (Liu et al., 2012). We employed root wounding–immersion, and bleaching occurred in eggplants after 29 days and spread from leaf veins to the surrounding tissue (**Figure 2D**). The silencing rate was 23% (**Supplementary Table S3**). *A. thaliana* is a model plant in the Brassicaceae family, and VIGS has been used for many gene functional studies (Senthil-Kumar and Mysore, 2011b). Bleaching occurred on day 5 after inoculation, and the silencing rate was 75%. Even though the root wounding–immersion method is suitable for *A. thaliana*, the survival rate was 48%, which is lower than that in the tomatoes, peppers, and eggplants. This may be because *A. thaliana* itself is weak, small, and non-viable when it is transplanted after root wounding–immersion treatment.

Under portable light excitation via LUYOR-3410RB illumination, pTRV2-*GFP* moved from the roots to the stems and leaves of the tomatoes, peppers, eggplants, and *A. thaliana* (**Figures 2G–J**). Moreover, we used root wounding–immersion on cabbage and cucumbers, but photobleaching was not observed (**Appendix 1**).

### 3.3 Successful application of root wounding–immersion in multiple plants

**Figure 3:**
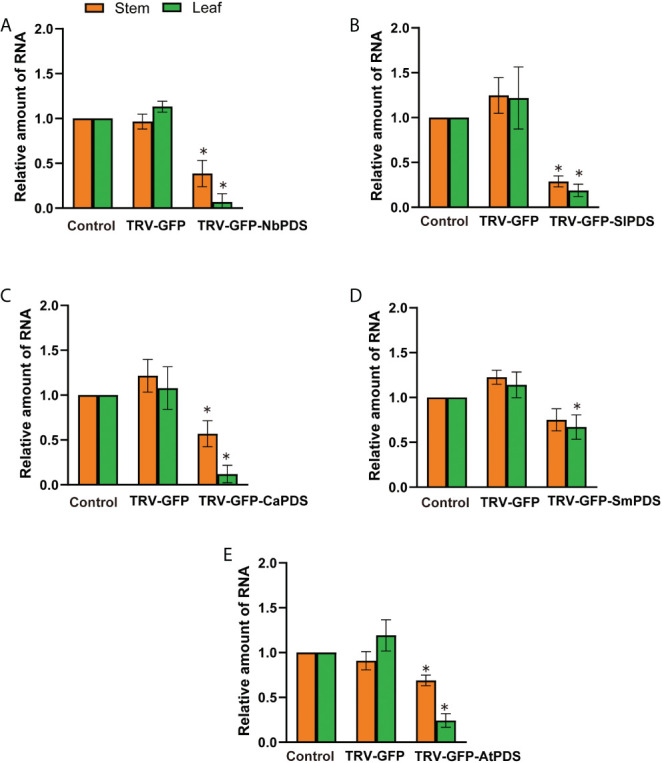
Virus-induced gene silencing in different plants through the root-soaking method. **(A–E)** Real-time fluorescence quantitative PCR measurement of *PDS* expression pattern in *Nicotiana benthamiana*, tomato, pepper, eggplant, and *A. thaliana*. The control expression level was assigned a value of 1. The error bars are the standard deviations of biological triplicates. The asterisk (*) means *P* < 0.05, based on Student’s *t*-test.

**Figure 4:**
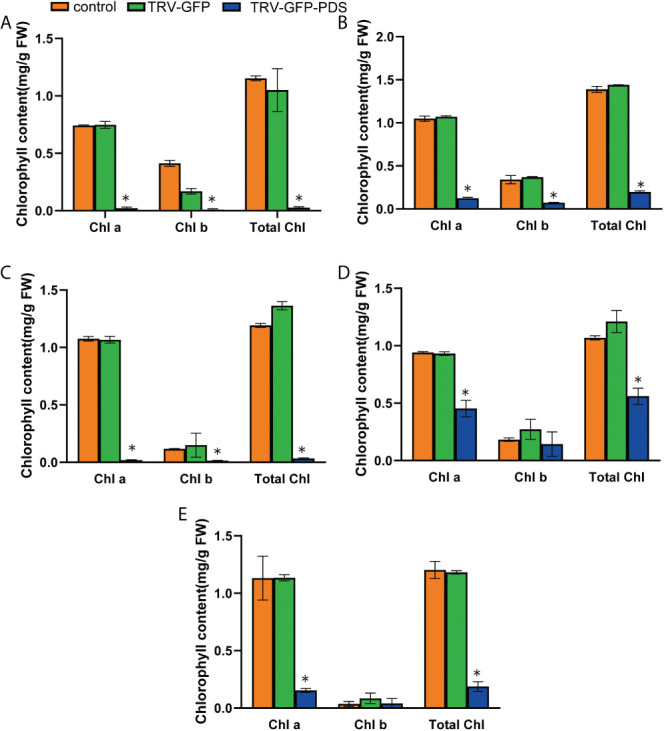
The VIGS system established through the root-soaking method resulted in a decrease in the chlorophyll content of plant leaves. **(A–E)** The decreases in chlorophyll a, chlorophyll b, and total chlorophyll contents in *Nicotiana benthamiana*, tomato, pepper, eggplant, and *A. thaliana* TRV2-*PDS*-infected plant leaves were measured. The error bars are the standard deviations of biological triplicates. The asterisk (*) means *P* < 0.05, based on Student’s *t*-test.

We used RT-qPCR to confirm *PDS* silencing at the molecular level, and showed that the average relative expression level of *PDS* was significantly lower in photobleached *N. benthamiana*, tomato, pepper, eggplant, and *A. thaliana* than in the control group (**Figures 3A–E**). The leaves became white owing to carotenoid deficiency and the photooxidative destruction of chlorophyll after *PDS* silencing (Kumagai et al., 1995). We measured chlorophyll a, chlorophyll b, and total chlorophyll contents upon the silencing of *N. benthamiana*, tomato, pepper, eggplant, and *A. thaliana*. The chlorophyll a, chlorophyll b, and total chlorophyll contents of *N. benthamiana*, tomato, eggplant, pepper, and *A. thaliana* TRV2-*GFP*-*PDS*-silenced plants decreased by more than 40% compared with those of the control group; specifically, the chlorophyll a, chlorophyll b, and total chlorophyll contents in *N. benthamiana* leaves decreased by more than 90% (**Figures 4A–E**).

**Figure 5:**
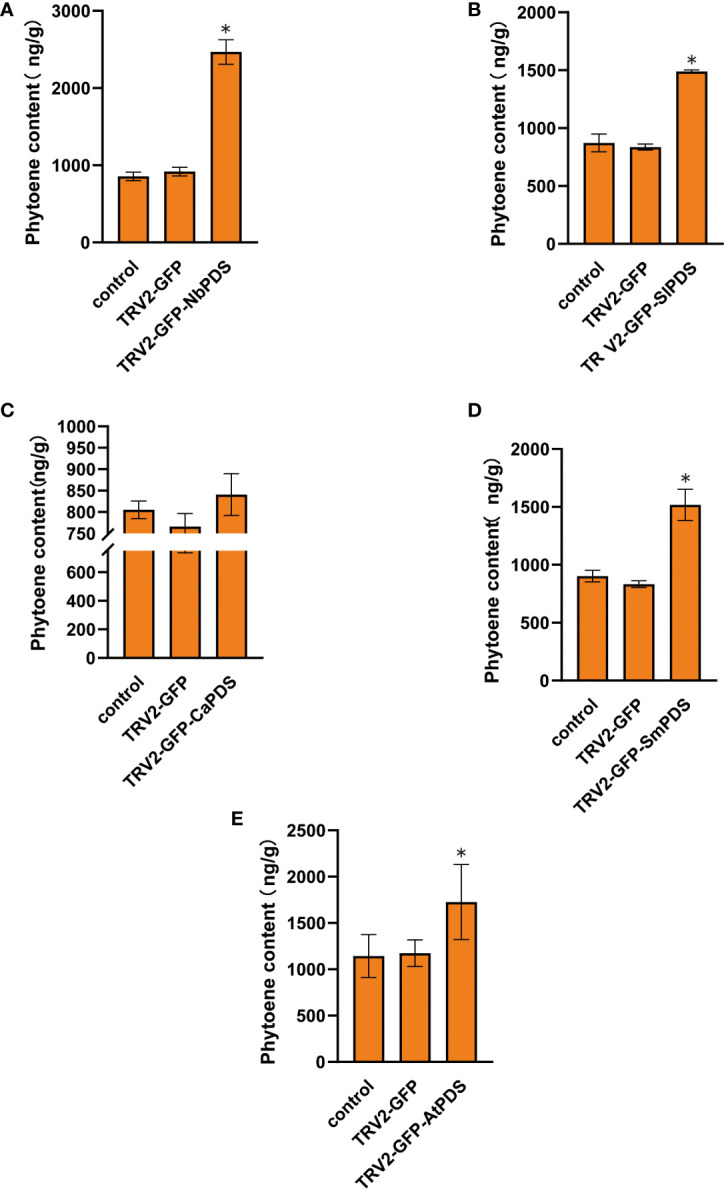
The VIGS system established through the root-soaking method resulted in an increase in the phytoene content of plant leaves. **(A–E)** The phytoene accumulation levels in *Nicotiana benthamiana*, tomato, pepper, eggplant, and *A. thaliana* TRV2-*PDS* infected plant leaves were measured. The error bars are the standard deviations of biological triplicates. The asterisk (*) means *P* < 0.05, based on Student’s *t*-test.

In transfected plants, *PDS* silencing inhibited the synthesis of carotenoids downstream of phytoene and caused high levels of phytoene (Kumagai et al., 1995; Fu et al., 2006). We measured the phytoene levels of five plants and found that phytoene content increased in the silenced plants (**Figures 5A–E**). These results confirm that root wounding–immersion can cause the *PDS* gene to be successfully silenced.

### 3.4 Application of root wounding–immersion in disease resistance

**Figure 6:**
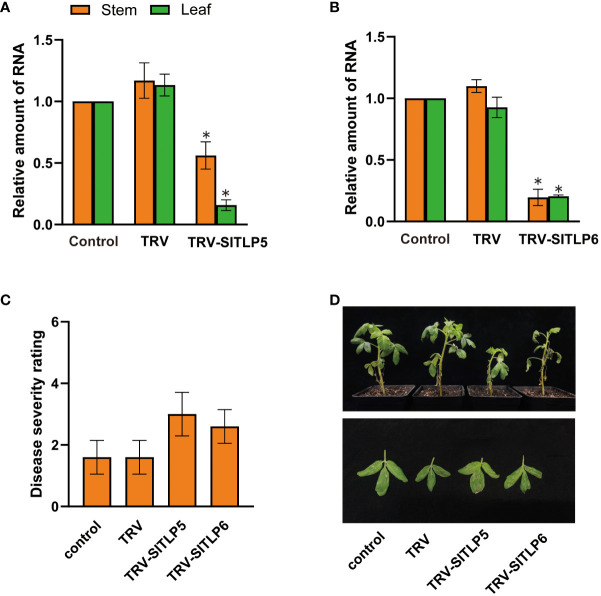
The VIGS system established using the root-soaking method reduced the disease resistance of tomato plants. **(A, B)** Real-time fluorescence quantitative PCR measurement of *SITLP5* and *SITLP6* expression patterns in the stems and leaves of *SITLP5*- and *SITLP6*-silenced plants. The control expression level was assigned a value of 1. **(C, D)** Disease severity and resistance of SITLP5- and SITLP6-silenced plants 7 days after inoculation with pathogens. The error bars are the standard deviations of biological triplicates. The asterisk (*) means *P* < 0.05, based on Student’s *t*-test.

Inoculation of a leaf with a VIGS vector containing a gene fragment leads to the transmission of the virus throughout the entire plant (Burch-Smith et al., 2006b). To test the reliability of the root wounding–immersion method, we silenced two late blight (*P. Infestans*) resistance genes (*SITLP5* and *SITLP6)* in tomatoes in addition to the commonly reported genes chalcone synthase (*CHS*) (Spitzer et al., 2007), H subunit of magnesium protoporphyrin chelatase (*ChlH*) (Hiriart et al., 2002), and anthocyanidin synthase (*ANS*) (Senthil-Kumar et al., 2008). *SITLP5* and *SITLP6* overexpression and knockout increased and decreased late blight resistance, respectively, in tomatoes (Zhu et al., 2021). The CLN2037 tomato inbred line was generated by selective breeding from *Solanum pimpinellifolium* and contains late blight resistance genes. Thus, it is widely used in disease-resistance selective breeding and mining of disease resistance genes (Nowicki et al., 2012; Zhang et al., 2013). We used the root wounding–immersion method to inoculate TRV1:TRV2-*GFP*-*SITLP5* and TRV1:TRV2-*GFP*- *SITLP6* in tomato (CLN2037E), and disease symptoms were observed 7 days after *P. infestans* inoculation in *SITLP5*- and *SITLP6*-silenced plants using the method of Zhu et al. (2021). RT-qPCR was used to measure *SITLP5* transcript abundance in silenced plants, and the average relative expression level of *SITLP5* decreased by 43% and 84% in the stems and leaves, respectively, in CLN2037E. Moreover, *SITLP6* decreased by 80% and 79% in the stems and leaves, respectively (**Figures 6A, B**). The DSR of the control plant was 1.6, which was classified as high resistance. After *SITLP5* and *SITLP6* silencing, the plants showed decreased *P. infestans* resistance (**Figures 6C, D**), and the calculated DSRs were 3.0 and 2.6, respectively, which was considered moderate resistance. These results showed that the root wounding–immersion method is similarly suitable for TRV-VIGS in other biological studies.

### 3.5 The root wounding–immersion method can be used for large-scale, high-efficiency VIGS experiments in genetic studies

Initially, Ruiz et al. (1998) used mechanical friction to deliver potato virus X (PVX) RNA obtained from in vitro transcription to plants and successfully obtained an *NbPDS* gene silencing phenotype. However, this method is difficult and cumbersome, and the silencing efficiency is low. Later, stem scratch and agroinfiltration methods were successfully applied to PVX VIGS (Bhaskar et al., 2009; Du et al., 2014). Antiviral silencing in some susceptible Solanaceae roots strongly inhibited PVX replication levels (Andika et al., 2015). Ratcliff et al. (2001) silenced target genes by injection infiltration and confirmed that TRV has many advantages over PVX, TMV, and TGMV. The TRV vector is able to invade roots and express GFP efficiently, whereas the widely used PVX vector is not (MacFarlane and Popovich, 2000). Bond and Baulcombe (2015) used TRV as a VIGS-RdDM silencing vector tool to augment the production of a 24-nt sRNA. The silencing rate of tomatoes when injection is used is 50% (Liu et al., 2002b). Spraying is also widely used for tomatoes, with a silencing rate of 90% (Liu et al., 2002a; Dinesh-Kumar et al., 2003). However, it has many equipment requirements, preventing many laboratories from employing this method. We developed the root wounding–immersion method and optimized it. We selected 3-week-old seedlings, removed 1/3 of the root lengthwise, and then immersed it in TRV1 and TRV2 *Agrobacterium* mixtures for 30 min. The *N. benthamiana* silencing rate was 95–100%. The root wounding–immersion method has similar strengths as using agrodrench. Agrodrench (Ryu et al., 2004) is a 1:1 mixture of *Agrobacterium* sp. TRV1 and TRV2 that is used to soak the roots, and a 10-mL pipette is then used to aspirate 3–5 mL into the crown of the plant. Although this method is simple and fast, it consumes a substantial amount of materials. After the TRV1 and TRV2 mixtures are prepared for the root wounding–immersion method, they can be used to immerse hundreds of plants each time. After immersion, a fresh bacterial suspension can be used repeatedly, which can save considerable time and costs. Moreover, 60–70% of Solanaceae plants other than *N. benthamiana* have shown the *PDS* silencing phenotype when agrodrench was used. The silencing rate of tomatoes when root wounding–immersion was used was 95%. In addition to Solanaceae plants, we found that the root wounding–immersion method is similarly suitable for *A. thaliana*.

## 4 Conclusion

In the post-genomic era, the large-scale functional genomics method is essential for converting sequence information to functional information. No recent breakthroughs have been achieved regarding TRV-VIGS. Thus, we developed the root wounding–immersion method and optimized the procedure. This is an extremely simple and efficient VIGS method that can be used to process large batches of plants (**Figures 1B, C**). We selected *PDS* and *GFP* as reporter genes and successfully silenced *N. benthamiana*, tomato, pepper, eggplant, and *A. thaliana*. In particular, the efficiency in *N. benthamiana* and tomato was 95–100%. Then, two resistance genes (*SITL5* and *SITL6*) were used to test the reliability of the root wounding–immersion method. In contrast to leaf infiltration, spray inoculation, and the use of agrodrench, the root wounding–immersion method does not require infiltration of individual leaves or preparation of large amounts of inoculation solution, and it has low laboratory requirements. VIGS experiments can be carried out in large batches and with high efficiency within 30 min. In addition, our method is suitable for plant species that are susceptible to root inoculation and seedling inoculation in early growth stages.

## Data availability statement

The original contributions presented in the study are included in the article/**Supplementary Material**, further inquiries can be directed to the corresponding author/s.

## Author contributions

XL: Writing – original draft, Writing – review & editing. NT: Writing – original draft. BX: Investigation, Writing – original draft. JX: Funding acquisition, Writing – review & editing. ZY: Funding acquisition, Writing – review & editing. CJ: Methodology, Writing – review & editing. YZ: Data curation, Writing – original draft. MD: Funding acquisition, Project administration, Writing – original draft. JL: Conceptualization, Funding acquisition, Investigation, Writing – review & editing. KZ: Formal analysis, Investigation, Methodology, Resources, Writing – review & editing.
