# Supplementary figures and images for: Establishment and application of a root wounding–immersion method for efficient virus-induced gene silencing in plants
Source: Front Plant Sci. 2024 Apr 19;15:1336726. doi: 10.3389/fpls.2024.1336726 (PMC11066161; doi:10.3389/fpls.2024.1336726)

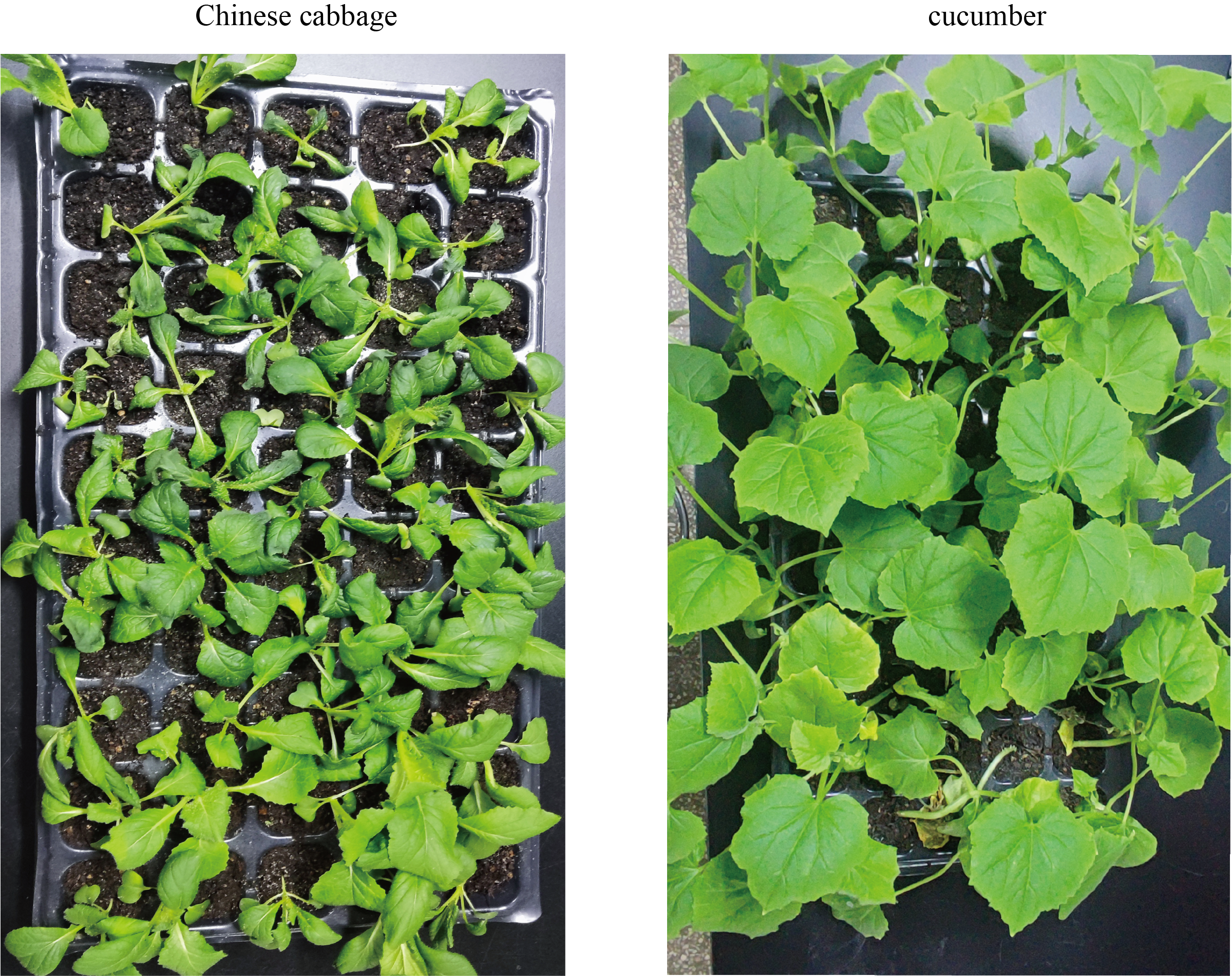

Supplement: Supplementary file 1 [file Image_1.png]
